# Multi-omics reveals total flavones from Abelmoschus manihot (L.) Medik. [Malvaceae] ameliorate MAFLD via PI3K/AKT/mTOR-mediated autophagy

**DOI:** 10.3389/fphar.2025.1601707

**Published:** 2025-07-11

**Authors:** Chao Lv, Lei Zhao, Jiani Hou, Hongyin Sun, Zhongsha Li, Yuesong Wu, Peizheng Shi, Yaping Xiao, Yunjin Xie, Wei Su, Mingzhu Yin

**Affiliations:** ^1^ Clinical Research Center, Medical Pathology Center, Cancer Early Detection, and Treatment Center and Translational Medicine Research Center, Chongqing University Three Gorges Hospital, Chongqing University, Chongqing, China; ^2^ Internal Medicine of Traditional Chinese Medicine, Chongqing University Three Gorges Hospital, Chongqing, China; ^3^ Chongqing Technical Innovation Center for Quality Evaluation and Identification of Authentic Medicinal Herbs, Chongqing, China; ^4^ Chongqing University Three Gorges Hospital and Academy for Advanced Interdisciplinary Technology, CQU-Ferenc Krausz Nobel Laureate Scientific Workstation, Chongqing, China; ^5^ School of Medicine, Chongqing University, Chongqing, China; ^6^ College of Elementary Education, Chongqing Preschool Education College, Wanzhou, Chongqing, China; ^7^ Department of Pathology and Pathophysiology, School of Basic Medical Sciences, Guangzhou University of Chinese Medicine, Guangzhou, China; ^8^ Department of Pharmacy and Pharmacology, Chongqing University Three Gorges Hospital, Chongqing, China

**Keywords:** total flavonoids from *Abelmoschus manihot* (L.) Medik. [Malvaceae], metabolic associated fatty liver disease, PI3K/AKT/mTOR pathway, autophagy, inflammation, oxidative stress

## Abstract

**Introduction:**

Metabolic-associated fatty liver disease (MAFLD) has emerged as a global health crisis, which is characterized by hepatic lipid accumulation, inflammation, and fibrosis. Currently, effective therapeutic strategies for MAFLD are still scarce.

**Methods:**

This study aimed to explore the hepatoprotective effects and underlying mechanisms of total flavones from Abelmoschus manihot (L.) Medik. (Malvaceae), abbreviated as TFA, in the context of MAFLD. Ultra-high-performance liquid chromatography-quadrupole orbitrap mass spectrometry (UHPLC-QTOF-MS) was used to identify the metabolites in TFA. MAFLD mice induced by a high-fat diet were treated with TFA at doses of 50 and 100 mg/kg. Body weight gain, hepatic lipid accumulation, and serum levels of alanine transaminase (ALT), aspartate transaminase (AST), total cholesterol (TC), and triglycerides (TG) were determined. Histological analysis was performed to evaluate hepatic steatosis, fibrosis, as well as the levels of inflammatory cytokines (IL-6, TNF-α) and antioxidant markers (SOD, GSH). Transcriptomic and metabolomic analyses were carried out to explore the molecular mechanisms. In vitro studies were conducted in HepG2 cells, and the role of autophagy was investigated using the autophagy inhibitor 3-MA.

**Results:**

Using UHPLC-QTOF-MS, 56 metabolites were identified in TFA, including hyperoside, rutin, and quercetin derivatives, which possess anti-lipidemic and anti-inflammatory properties. In MAFLD mice, TFA treatment significantly decreased body weight gain, hepatic lipid accumulation, and the serum levels of ALT, AST, TC, and TG. Histological analysis demonstrated that TFA alleviated hepatic steatosis and fibrosis, with decreased levels of inflammatory cytokines and increased antioxidant markers. Transcriptomic and metabolomic analyses indicated that TFA regulated nucleotide metabolism, pyrimidine metabolism, and the PI3K/AKT/mTOR signaling pathway. In HepG2 cells, TFA inhibited palmitic acid/oleic acid-induced lipid deposition and the production of reactive oxygen species (ROS). Mechanistically, TFA activated autophagy through the inhibition of PI3K/AKT/mTOR phosphorylation, as demonstrated by the increased LC3II/I conversion and decreased p62 expression. The autophagy inhibitor 3-MA abolished the protective effects of TFA.

**Discussion:**

Our findings suggest that TFA ameliorates MAFLD via promoting PI3K/AKT/mTOR-mediated autophagy. The metabolites identified in TFA might contribute to its multi-target therapeutic effects. Considering the limited treatment options for MAFLD, TFA exhibits great potential as a novel therapeutic agent for MAFLD intervention, thus justifying further preclinical and clinical investigations.

## 1 Introduction

Metabolic Associated Fatty Liver Disease (MAFLD) has emerged as a global public health crisis, with a prevalence rate affecting nearly 25% of the global population (Miao et al., 2024; Cotter and Rinella, 2020). Furthermore, epidemiological studies have documented a consistent upward trend in MAFLD incidence rates worldwide (Younossi et al., 2023). MAFLD represents a complex clinicopathological syndrome characterized by excessive hepatic lipid accumulation, independent of alcohol consumption and other established hepatotoxic factors. Without timely intervention, MAFLD progression may culminate in severe complications including cirrhosis and hepatocellular carcinoma (Pais et al., 2016). Importantly, MAFLD has been identified as a significant risk factor for multiple systemic disorders, including type 2 diabetes mellitus, cardiovascular diseases, and chronic kidney disease (Byrne and Targher, 2015). Despite extensive research efforts, there remains an unmet clinical need for effective therapeutic strategies against MAFLD. Compounding this challenge, emerging evidence reveals the multifactorial pathogenesis of MAFLD, presenting significant obstacles for targeted drug development. Notably, natural metabolites have garnered increasing scientific interest for their potential hepatoprotective effects in MAFLD management (Yang et al., 2024; Sun et al., 2023a; Liu et al., 2025).

Flavonoids represent a ubiquitous class of phytochemicals distributed across various plant species, with particularly high concentrations in medicinal botanical drugs. Structural analysis reveals significant heterogeneity in flavonoid metabolites derived from distinct botanical sources. The structural complexity of flavonoids poses significant challenges in isolating individual metabolites with high purity. Consequently, researchers typically work with total flavonoid extracts containing multiple metabolites in varying proportions. Natural flavonoids demonstrate significant hepatoprotective efficacy, making them promising candidates for managing metabolic disorders. The hepatoprotective mechanisms primarily involve modulation of hepatic lipid metabolism through enhanced hyperlipolysis and fibrotic lipolysis, thereby mitigating oxidative stress-induced hepatocyte damage (Li et al., 2021; Wang et al., 2021; Fan et al., 2023a). Furthermore, flavonoids exhibit pleiotropic effects including anti-inflammatory, antioxidant, anti-apoptotic, and immunomodulatory properties (Lv et al., 2023; Ku et al., 2020). Extensive preclinical studies have consistently validated the hepatoprotective efficacy of flavonoid metabolites. However, the precise molecular mechanisms underlying flavonoid-mediated hepatoprotection remain incompletely characterized, warranting further investigation.

Autophagy represents an essential cellular catabolic process that critically regulates cellular homeostasis and functional integrity. Experimental evidence consistently demonstrates that impaired hepatocyte autophagy leads to marked intracellular lipid accumulation, independent of nutritional status (Shen et al., 2023; Gao et al., 2020). Conversely, chronic high-fat diet exposure induces progressive suppression of hepatic autophagy, culminating in hepatocyte injury and pathological steatosis. The PI3K/AKT/mTOR signaling axis has emerged as a central regulator of autophagic flux, attracting substantial research focus. mTOR inhibition triggers GSK-3β-mediated phosphorylation and activation of ULK1, initiating autophagosome formation. Pharmacological inhibition of PI3K/AKT/mTOR phosphorylation potently induces autophagic activity (Liu et al., 2020a; Sun et al., 2023b). This cascade consequently alleviates hepatocyte lipid overload and inflammatory signaling, thereby mitigating MAFLD pathogenesis.

*Abelmoschus manihot (L.) Medik. [*Malvaceae*]* a pharmacologically active botanical drug in traditional Chinese medicine, has been widely utilized for its anti-inflammatory properties in clinical practice. Phytochemical analysis has identified total flavones of *A. manihot (L.) Medik. [*Malvaceae*]* (TFA) as the principal bioactive metabolites responsible for its therapeutic effects (Xue et al., 2023). Although TFA has demonstrated therapeutic potential in various metabolic and inflammatory disorders, its specific role in MAFLD remains unexplored (Diao et al., 2023; Tao et al., 2024; Zhou et al., 2022). Given the rising global prevalence of MAFLD and the limited efficacy of current treatment options, investigating TFA’s effects on this condition could offer novel mechanistic insights and therapeutic opportunities. This investigation commenced with comprehensive phytochemical profiling to characterize the major metabolites of TFA. Through integrated *in vivo* and *in vitro* experimental approaches, we elucidated the hepatoprotective mechanism of TFA through PI3K/AKT/mTOR-mediated autophagic regulation in MAFLD. These findings establish TFA as a promising therapeutic candidate with translational potential for MAFLD intervention.

## 2 Materials and methods

### 2.1 Sample preparation

*Abelmoschus manihot (L.) Medik*. *[*Malvaceae*]* was collected from Wuxi County, Chongqing, China. The extraction of TFA from its flowers was conducted by the Department of Pharmacy at Chongqing University, China. The extraction of TFA was conducted through an optimized protocol combining high-speed homogenization with ultrasound-assisted extraction, a dual-phase approach that maximizes flavonoid yield while maintaining structural integrity of bioactive metabolites. The crude extract was subsequently purified using AB-8 macroporous adsorption resin, a weakly polar resin known for its efficacy in removing impurities and enriching target metabolites. The purification process consisted of adsorption and desorption steps to effectively isolate and concentrate the total flavonoids. The flavonoid-enriched solution was then concentrated under reduced pressure and dried to yield a fine, yellowish powder. The resulting powder, representing the purified total flavonoids, was stored at 4°C for subsequent analysis and experimental use. The entire extraction and purification procedure was meticulously optimized to ensure high purity and yield of the total flavonoids, which is crucial for subsequent biological and pharmacological studies.

### 2.2 TFA metabolite testing

Precisely 20.6 mg of powdered TCM sample was homogenized with 1.00 mL of 50% methanol in a 2 mL polypropylene centrifuge tube, followed by ultrasonication for 30 min. A 500 μL aliquot of the suspension was transferred to a pre-chilled 1.5 mL microcentrifuge tube and centrifuged at 4°C (12,000 rpm) for 10 min using a refrigerated centrifuge. The clarified supernatant (100 μL) was carefully transferred using a calibrated micropipette into a certified LC-MS injection vial with 250 μL glass insert.

Chromatographic separation was performed on a Vanquish UHPLC system (Thermo Fisher Scientific, Inc., Waltham, MA, United States) with ACQUITY UPLC^®^ HSS T3 column (2.1 × 100 mm, 1.7 μm; Waters Corp., MA, United States). The mobile phase comprised (A) 0.1% formic acid and (B) acetonitrile at 0.3 mL/min flow rate, with column oven maintained at 40°C.

Mass spectrometry analysis was conducted on Q Exactive™ HF-X system (Q Exactive, Thermo Fisher Scientific, Inc., Waltham, MA, United States) with heated electrospray ionization (HESI-II) source. Ionization parameters: spray voltage 3.7 kV (positive)/3.5 kV (negative), capillary temp 320°C, sheath gas 30 psi, auxiliary gas 10 psi. High-purity nitrogen (99.999%) was used as sheath/auxiliary gas and collision gas (1.5 mTorr). Data acquisition was performed in “Full scan/dd-MS2” mode. Full scan parameters: resolution 7,000, AGC target 1 × 10^6^, max injection time 50 m. dd-MS2 parameters: resolution 17,500, AGC 1 × 10^5^, isolation window 2 m/z, stepped NCE 10/30/60 V, threshold 1 × 10^5^.

### 2.3 Animals and treatment

Male C57BL/6J mice (6–8weeks, 18–22g) were obtained from Jiangsu Huachuang Xinnuo Pharmaceutical Technology Co., Ltd. (Jiangsu, China). Following a 7-day acclimatization with standard chow in specific pathogen-free (SPF) conditions, mice were randomly divided into four groups (n = 8/group): Control, Model, TFA-L (50 mg/kg), and TFA-H (100 mg/kg). Control mice received normal chow diet (NCD), while other groups were fed high-fat diet (HFD; 60% fat, D12492) for 16 weeks. Control and Model groups received vehicle, whereas TFA-L and TFA-H groups were administered TFA via daily oral gavage. The study protocol was approved by the Institutional Animal Care and Use Committee of Chongqing University Three Gorges Hospital (IACUC No. SXYYDW 2024-088).

### 2.4 Weight and biochemical analysis

Weekly body weight measurements were recorded using an electronic balanceby investigators blinded to group assignments. Following 12-h fasting (water *ad libitum*), mice were anesthetized with 2% isoflurane for terminal blood collection via cardiac puncture and liver tissue harvesting, which were immediately snap-frozen in liquid nitrogen. Serum biochemical parameters including TC (A111-2-1), TG (A110-1-1), LDL-C (A113-2-1), ALT (C009-2-1), and AST (C010-2-1) were quantified using commercial kits (Nanjing Jiancheng Bioengineering Institute) according to manufacturer’s protocols.

### 2.5 Cell culture and treatment

HepG2 cells were maintained in DMEM (C11995500BT, Gibco) supplemented with 10% FBS (FSP500, ExCell Bio) and 1% penicillin-streptomycin (15,140,122, Gibco) at 37°C in a humidified 5% CO_2_ incubator. For *in vitro* MAFLD modeling, cells were exposed to 0.25 mM palmitic acid (PA) and 0.5 mM oleic acid (OA) (KT004, Kunchuang) for 24 h (Wu et al., 2024). TFA stock solution (100 mg/mL in DMEM) was prepared and stored at 4°C. Following MAFLD induction, cells were co-treated with TFA and PA/OA mixture for 24 h to assess therapeutic effects.

### 2.6 TFA cytotoxicity analysis in HepG2 cells

Cytotoxicity assessment was performed using Cell Counting Kit-8 (CCK-8, Dojindo Laboratories) according to the manufacturer’s protocol. HepG2 cells were seeded at 3 × 10^3^ cells/well in 96-well plates and cultured for 24 h to achieve 70%–80% confluence. Cells were treated with TFA (0–16 mg/mL) in serum-free medium for 24 h. Following treatment, 10 μL CCK-8 reagent was added per well and plates were incubated at 37°C, 5% CO_2_ for 4 h. Absorbance was measured at 450 nm using a microplate reader.

### 2.7 Hematoxylin and eosin (H&E) staining

Mice liver tissues were fixed in 4% paraformaldehyde and then embedded in paraffin. Paraffin-embedded tissues were sectioned at 5 μm thickness using a rotary microtome (Leica RM2235) for hematoxylin-eosin (H&E) staining. Tissue sections were deparaffinized in xylene (2 × 5 min) and rehydrated through graded ethanol series (100%, 95%, 80%, 70%, each for 2 min). Sections were stained with Mayer’s hematoxylin (5 min) followed by eosin Y (2 min) with intermediate washes in distilled water. Stained sections were dehydrated through ascending ethanol series (70%, 80%, 95%, 100%, each for 2 min) and cleared in xylene (2 × 5 min). Finally, the sections were mounted with neutral balsam and observed under a microscope for histological analysis.

### 2.8 RNA-sequencing analysis

Whole transcriptomics sequencing was performed using RNA sequencing technology. Total RNA was isolated from liver tissue using TRIzol Reagent (Thermo Fisher) followed by quality assessment with NanoDrop One (A260/A280 = 1.8–2.2). Sequencing libraries were prepared from 2 μg total RNA using NEBNext^®^ Ultra II reagents, with 200–500 bp inserts selected by 0.6×AMPure XP bead purification prior to Illumina HiSeq X Ten sequencing. Raw reads were processed through HISAT2 alignment and featurecounts quantification. Differentially expressed genes were identified with |log2FC|≥0.25 and FDR-adjusted *p* < 0.05 using DESeq2.

### 2.9 Liver untargeted metabolomics analysis

Liver samples underwent preprocessing through sequential metabolite extraction and purification, ensuring stability and representativeness of biological specimens. High-resolution liquid chromatography-mass spectrometry (LC-MS) was subsequently implemented to systematically characterize the metabolic profiles. Data-dependent acquisition (DDA) was employed to capture metabolite-specific fragmentation patterns, utilizing m/z differentials to generate paired primary and secondary mass spectra. Multivariate chemometric analyses—including principal component analysis (PCA) and partial least squares-discriminant analysis (PLS-DA)—were conducted for data processing, quality control validation, and statistical evaluation. This analytical framework enabled identification of group-specific metabolite variations, pathway enrichment analysis (KEGG database), and mechanistic exploration of hepatic pathophysiology.

### 2.10 Oil Red O staining

HepG2 cells from experimental groups were sequentially washed thrice with phosphate-buffered saline (PBS) and fixed with 4% paraformaldehyde at room temperature for 15 min. Fixed cells were subsequently incubated with freshly prepared Oil Red O solution for 30 min at room temperature to visualize lipid accumulation. Sequential graded washes were performed using 60% isopropanol (3 × 5 min) followed by PBS hydration (3 × 5 min) to remove unbound dye. For microscopic analysis, adherent cells were imaged using an optical microscope Leica DMi8. Lipid accumulation was quantified by threshold-based area segmentation in ImageJ with normalization to total cellular area, employing blinded analysis protocol.

### 2.11 ROS detection

HepG2 cells were seeded at 5 × 10^5^ cells/well in 2 mL complete DMEM using 6-well culture plates and allowed to adhere for 12 h under standard conditions (37°C, 5% CO_2_). Cells were exposed to predetermined interventions for 24 h. Cellular ROS levels were detected by loading 5 μM dihydroethidium (DHE) probe (diluted in serum-free medium) followed by 30 min incubation in darkness (CA1420, Solarbio). Post-staining, cells were subjected to three PBS washes (3 × 5 min) prior to fluorescence imaging using an Thermo M5000.

### 2.12 Determination of SOD and GSH

Cellular lysates were prepared by homogenization in ice followed by centrifugation (12,000 g, 10 min, 4°C) with subsequent collection of supernatant aliquots (200 μL) avoiding pellet contamination. Superoxide dismutase (SOD) activity and glutathione (GSH) levels were quantified using commercial assay kits (BC5165 and BC1175, respectively; Solarbio) following the manufacturer’s protocols, with all measurements performed in triplicate.

### 2.13 Cellular autophagy vesicle assay

HepG2 cells in the logarithmic growth phase were plated in 6-well plates at a density of 3 × 10^5^ cells/well and maintained for 24 h. Following 24 h treatment with various TFA concentrations, autophagy was evaluated using the Autophagy Staining Assay Kit (Beyotime, C3018S). The kit’s fluorescent probe, monodansylcadaverine (MDC), facilitated efficient autophagy detection. Fluorescence microscopy images were acquired and analyzed quantitatively for fluorescence intensity using ImageJ.

### 2.14 Enzyme linked immunosorbent assay (ELISA)

Cytokine quantification was performed using commercially available ELISA kits (MultiSciences Biotech Co.) for interleukin-6 (IL-6, Cat# EK206) and tumor necrosis factor-α (TNF-α, Cat# EK282), following manufacturer-recommended protocols. Briefly, 50 μL of appropriately diluted samples were dispensed into pre-coated 96-well microplates in triplicate. Following sample addition, 100 μL of horseradish peroxidase (HRP)-conjugated detection antibody was incubated with the samples at 37°C for 60 min in a humidified chamber. Post-substrate incubation, absorbance was measured at 450 nm using a SpectraMax i3x.

### 2.15 Real-time quantitative PCR analysis

Total RNA was isolated from mouse liver tissue and HepG2 cells using Trizol reagent (Cat. No. 15596026, Invitrogen) according to the manufacturer’s instructions. Genomic DNA was eliminated from the RNA samples using DNase I, followed by reverse transcription to cDNA using the High-Capacity cDNA Reverse Transcription Kit (Vazyme, R323-01). Relative mRNA expression levels were quantified using the QuantStudio 5 Real-Time PCR System (Thermo Fisher Scientific, United States) with PowerUp SYBR Green Master Mix (Vazyme, Q711-02). Custom-designed PCR primers (Shanghai Sangon Biotech Co., Ltd.) were used for qPCR, and their sequences are provided in Supplementary Table S1.

### 2.16 Western blot

Protein lysates were prepared from liver tissue and HepG2 cells using ice-cold RIPA buffer (Beyotime, P0013B) supplemented with protease inhibitor cocktail (Solarbio, 329–98-6) and phosphatase inhibitors (Selleck, B15001), with protein concentration determined by BCA assay (Beyotime, P0010). Equal protein aliquots (30 μg/lane) were resolved on 10% SDS-PAGE gels and electrotransferred to PVDF membranes (0.45 μm, IPVH00010, Millipore). Membranes were blocked, followed by overnight incubation with primary antibodies (4°C) and 1 h incubation with HRP-conjugated secondary antibodies, with detection using ECL substrate (BL520A, Biosharp) on a Bio-Rad ChemiDoc. Protein expression levels were normalized to GAPDH (internal control) using ImageJ for densitometric analysis. Primary antibodies were obtained as follows: PI3K (AF6241), p-PI3K (AF3241), AKT (AF6261), p-AKT (AF0016), LC-3B (AF4650), p-mTOR (AF3308), and p62 (AF5384) from Affinity Biosciences; IL-6 (BS6419) and TNF-α (BS 1857) from Bioworld Technology. Antibody dilutions were optimized as: goat anti-mouse IgG-HRP (1:2000), goat anti-rabbit IgG-HRP (1:2000), PI3K (1:1000), p-PI3K (1:1000), AKT (1:1000), p-AKT (1:1000), LC-3B (1:1000), p-mTOR (1:1000), p62 (1:1000), IL-6 (1:1000), and TNF-α (1:1000) in blocking buffer.

### 2.17 Data analysis

The results from each group are presented as mean ± standard deviation. Differences between two groups were analyzed using the t-test, while differences among multiple groups were assessed using one-way analysis of variance (ANOVA). Statistical analyses were performed using SPSS 22.0, with a significance level set at *P <* 0.05.

## 3 Results

### 3.1 Determination of the effective metabolites of TFA by UHPLC-QTOF-MS

**FIGURE 1:**
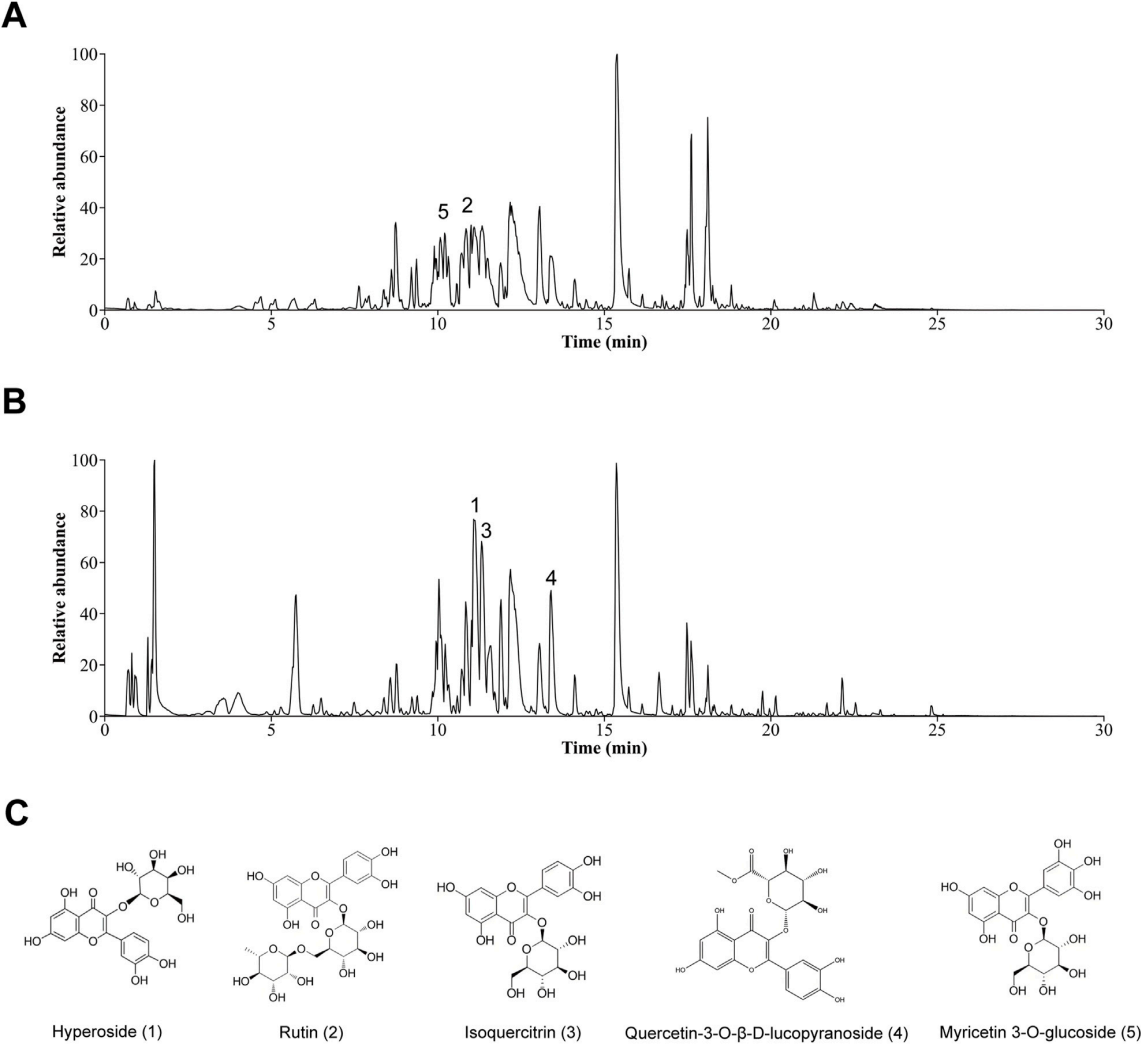
Graphs of TFA. **(A)** Plot of the basal peak of positive ion mode. **(B)** Plot of the basal peak of negative ion mode. **(C)** Structures of the five main chemical metabolites in TFA.

In this study, the TFA was extracted using high-speed homogenisation-ultrasonic-assisted liquid extraction and purified by removing impurities with AB-8 weakly polar macroporous adsorbent resin. The separated and enriched TFA concentrate was subsequently dried to obtain a brownish-yellow powder. As depicted in Figure 1; Supplementary Table S2, this powder was analyzed by ultra-high-performance liquid chromatography-quadrupole orbitrap high-resolution mass spectrometry (UHPLC-Q-Orbitrap HRMS). A total of 56 metabolites were detected and classified into flavonoids, coumarins and their derivatives, organooxygen compounds, prenol lipids, cinnamic acids and their derivatives, benzene and substituted derivatives. The main metabolites included hyperoside, rutin, isoquercitrin, quercetin-3-O-β-D-lucopyranoside, and myricetin 3-O-glucoside.

### 3.2 TFA improves body weight, lipid, and glucose metabolism in HFD-induced MAFLD mice

**FIGURE 2:**
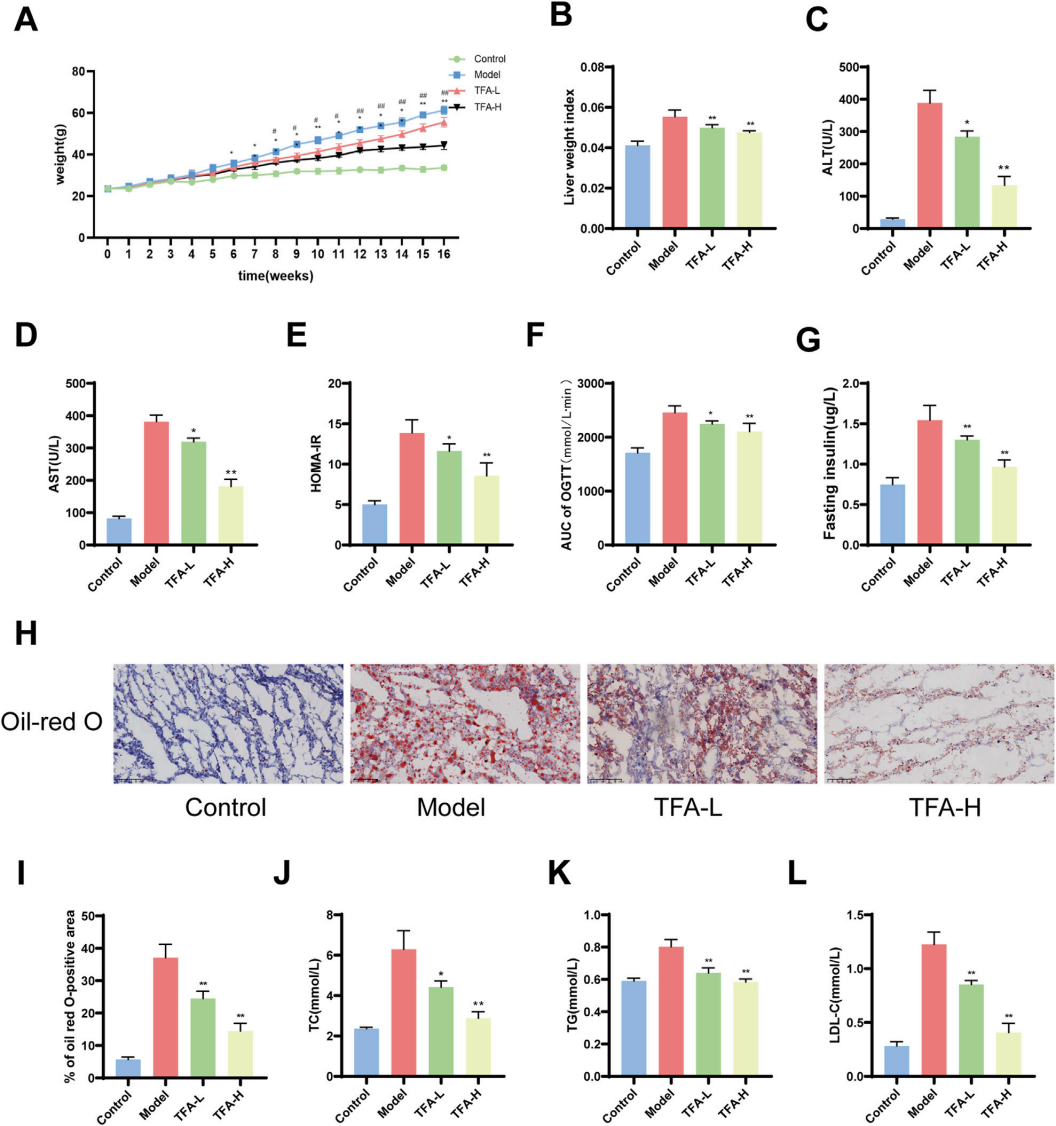
Effects of TFA on blood glucose and lipid levels in MAFLD mice. **(A)** Weekly body weight changes. **(B)** Liver weight index. **(C)** ALT levels. **(D)** AST levels. **(E)** Insulin resistance index. **(F)** Area under the curve for oral glucose tolerance test. **(G)** Fasting insulin levels. **(H)** Oil Red O staining of liver. **(I)** Percentage of areas stained positive for Oil Red O. **(J)** TC levels. **(K)** TG levels. **(L)** LDL-C levels. ^*^ *P <* 0.05 vs. Model. ^**^ *P <* 0.01 vs. Model.

To evaluate the impact of TFA on MAFLD, HFD-induced MAFLD mice were used in the experiment. The results showed significant differences in the body weight gain curves among the four groups. By the fifth week, the body weight of mice fed with HFD was significantly higher than that of mice fed with NCD. However, the weight of the HFD + TFA-L group was significantly reduced after 10 weeks of treatment (Figure 2A). At the same time, the liver mass index was significantly reduced by the TFA-L and TFA-H intervention (Figure 2B). To assess the efficacy of TFA-L and TFA-H in treating HFD-induced liver injury, the levels of serum ALT and AST were measured. The results revealed that the levels of serum ALT and AST were remarkably reduced after TFA administration (Figures 2C,D).

To assess the effect of TFA on lipid and glucose metabolism in MAFLD mice, the lipid and glucose levels in the serum and liver of the mice were measured. Intraperitoneal insulin tolerance test and intraperitoneal glucose tolerance test were employed to determine the effect of TFA on stabilizing insulin homeostasis. The results demonstrated that TFA-L and TFA-H treatment could improve insulin resistance in MAFLD mice (Figures 2E–G). Oil Red O staining of liver tissue revealed that both TFA-L and TFA-H treatment significantly reduced hepatic lipid accumulation in HFD-fed mice (Figures 2H,I). In addition, the results indicated that HFD feeding significantly increased the levels of TC and TG in the serum and liver, while TFA-L and TFA-H treatment significantly decreased the elevated TC and TG levels (Figures 2J,K). After 16 weeks of HFD treatment, the level of LDL-C in the serum was promoted to increase (Figure 2L). In summary, these results suggest that TFA improve systemic lipid and glucose metabolism in MAFLD mice in a dose-dependent manner.

### 3.3 TFA alleviates liver injury in MAFLD mice

**FIGURE 3:**
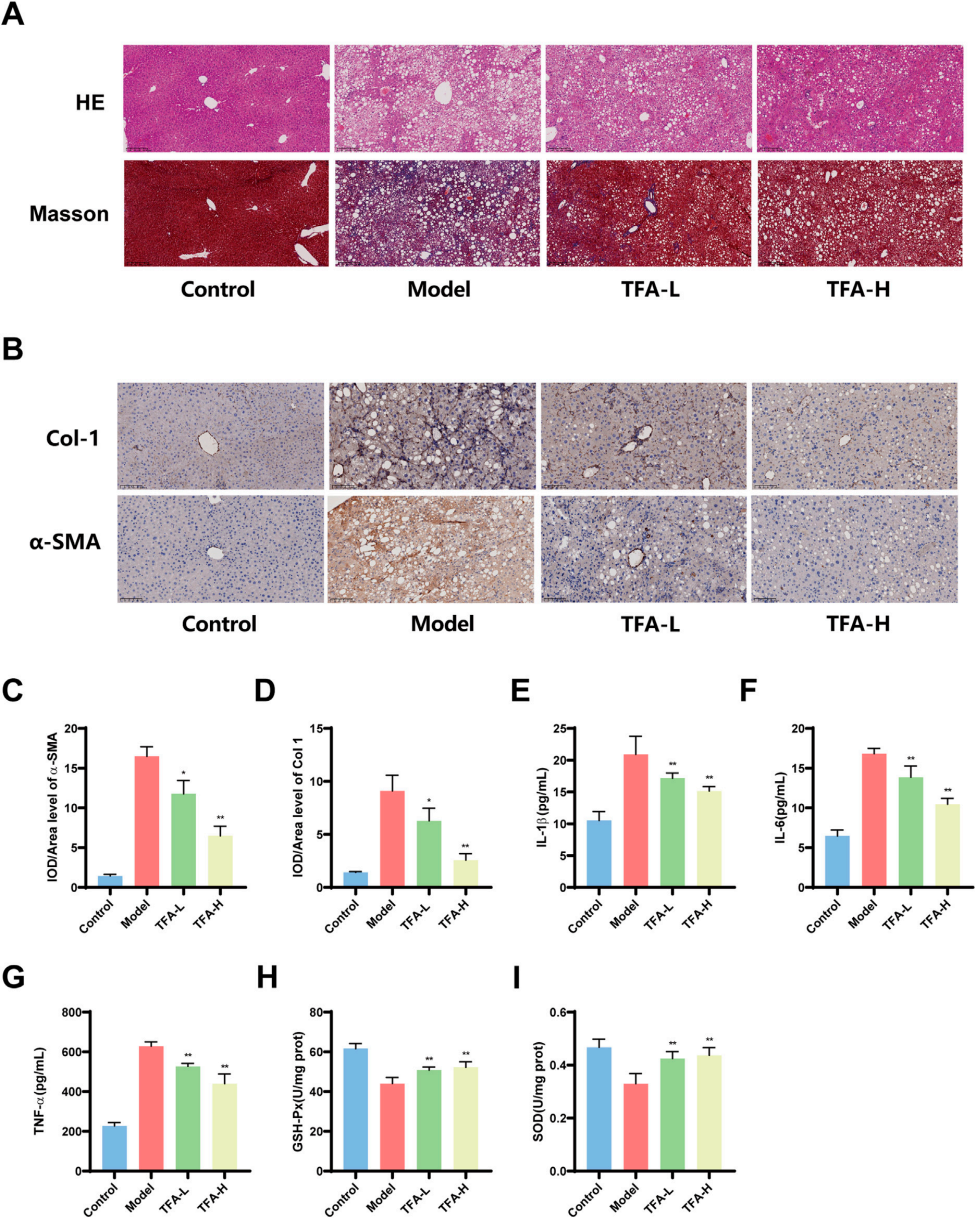
Effects of TFA on hepatic pathological injury, inflammatory levels, oxidative stress and fibrosis in MAFLD mice. **(A)** Representative images of HE and Masson staining of mice liver tissues. **(B)** IHC analysis of α-SMA and Col1 expression in mice liver tissues. **(C)** Quantification of α-SMA-positive area in liver tissues by IHC. **(D)** Quantification of Col1-positive area in liver tissues by IHC. **(E)** Hepatic IL-1β levels in mice. **(F)** Hepatic IL-6 levels in mice. **(G)** Hepatic TNF-α levels in mice. **(H)** Hepatic GSH-Px levels in mice. **(I)** Hepatic SOD levels in mice. ^*^ *P <* 0.05 vs. Model. ^**^ *P <* 0.01 vs. Model.

The liver injury in MAFLD is characterized by inflammation and fibrosis. To further evaluate the influence of TFA on hepatic steatosis and lipid accumulation in MAFLD mice, HE staining was utilized to evaluate the degree of steatosis within the liver tissue. The results indicated that following TFA-L and TFA-H treatment, the liver injury and hepatic steatosis in MAFLD mice were alleviated. Masson staining demonstrated the fibrosis of the liver tissue around the hepatic sinusoids in mice. After TFA-L and TFA-H treatment, the infiltration of inflammatory cells was reduced, and the liver fibrosis was ameliorated and was dose dependent (Figure 3A).

Additionally, immunohistochemical analysis demonstrated that TFA-L and TFA-H significantly decreased the expression of α-smooth muscle actin (α-SMA) and type I collagen (Col-1) in mice liver tissues, indicating its ability to inhibit the progression of hepatic fibrosis (Figures 3B–D). Excessive inflammatory and oxidative stress damage exacerbates the progression of MAFLD, leading to increased hepatocyte injury and potentially triggering more severe liver conditions. Further studies revealed that TFA-L and TFA-H significantly reduced the levels of hepatic inflammatory cytokines, including IL-1β, IL-6, and TNF-α, indicating its potent anti-inflammatory effects (Figures 3E–G). Concurrently, TFA-L and TFA-H treatment markedly elevated the levels of antioxidant stress markers, SOD and GSH, suggesting that TFA alleviates oxidative stress damage by enhancing antioxidant capacity (Figures 3H,I).

### 3.4 Metabolomic analysis of different metabolites in TFA-treated MAFLD livers

**FIGURE 4:**
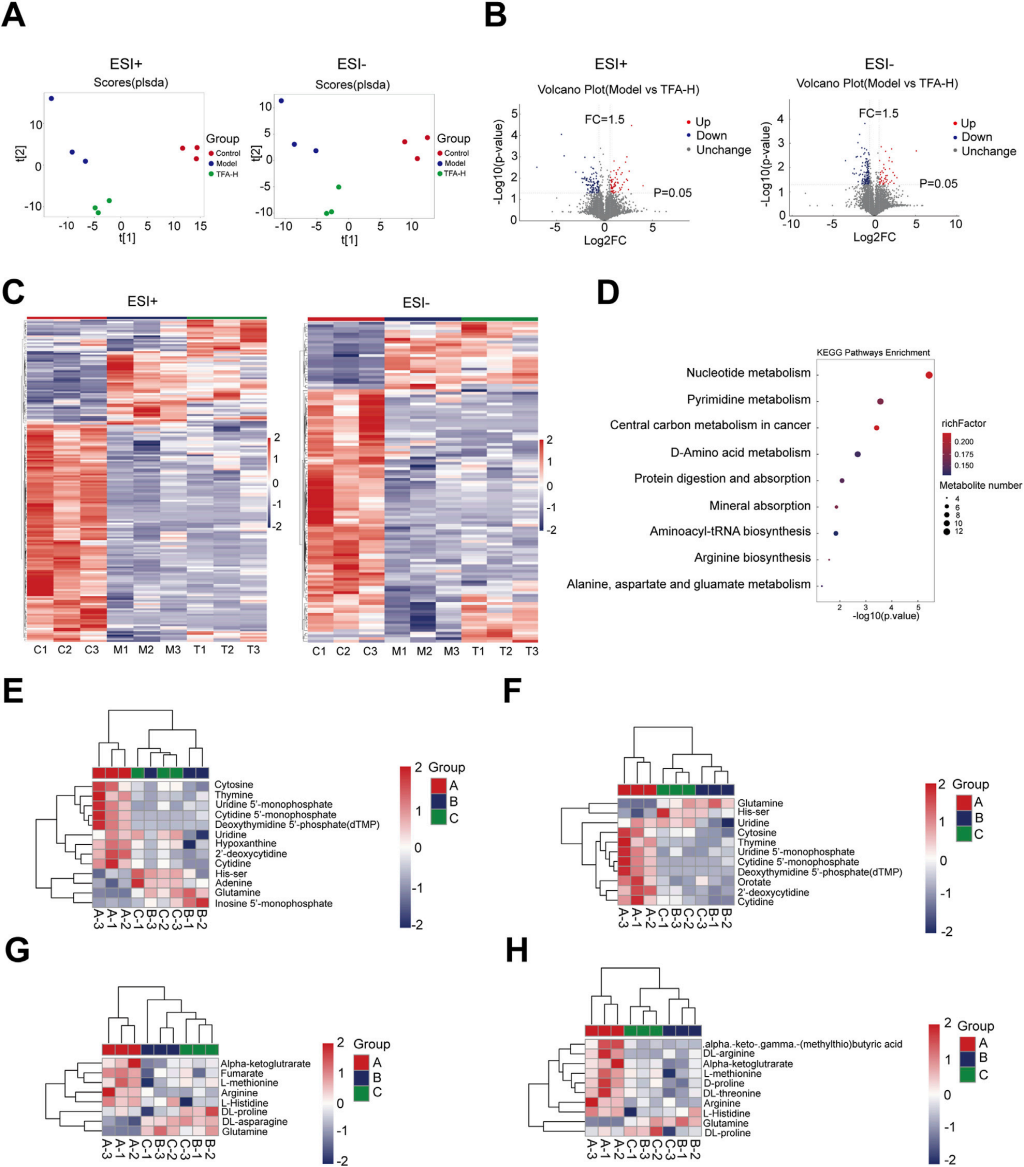
Analysis of differential metabolites of MAFLD by TFA. **(A)** OPLS-DA analyses with high experimental stability and reliability. **(B)** Differential metabolites between model and TFA-H groups. **(C)** Clustering heatmap of significant differential metabolites in each group. **(D)** KEGG enrichment analysis of differential metabolite metabolic pathways. **(E)** Nucleotide metabolism in each group. **(F)** Pyrimidine metabolism in each group. **(G)** Central carbon metabolism in cancer in each group. **(H)** D-Amino acid metabolism in each group.

This study employed untargeted metabolomics to analyze the hepatic metabolite profiles of mice in the control, model, and TFA-H-treated groups. OPLS-DA demonstrated high experimental stability and reliability (Figure 4A). Further analysis revealed that, following TFA-H treatment, 172 differential metabolites were identified in negative ion mode between the model and TFA-H groups, with 125 downregulated and 47 upregulated metabolites. In positive ion mode, 173 differential metabolites were identified, including 104 downregulated and 69 upregulated metabolites (Figure 4B). Additional analysis comparing the model and TFA-H groups identified differential metabolites between these two groups (Figure 4C). KEGG enrichment analysis revealed that the differential metabolites primarily affect Nucleotide metabolism, Pyrimidine metabolism, Central carbon metabolism in cancer and D-Amino acid metabolism (Figures 4D–H).

### 3.5 Transcriptomic analysis of differential genes in TFA-treated MAFLD livers

**FIGURE 5:**
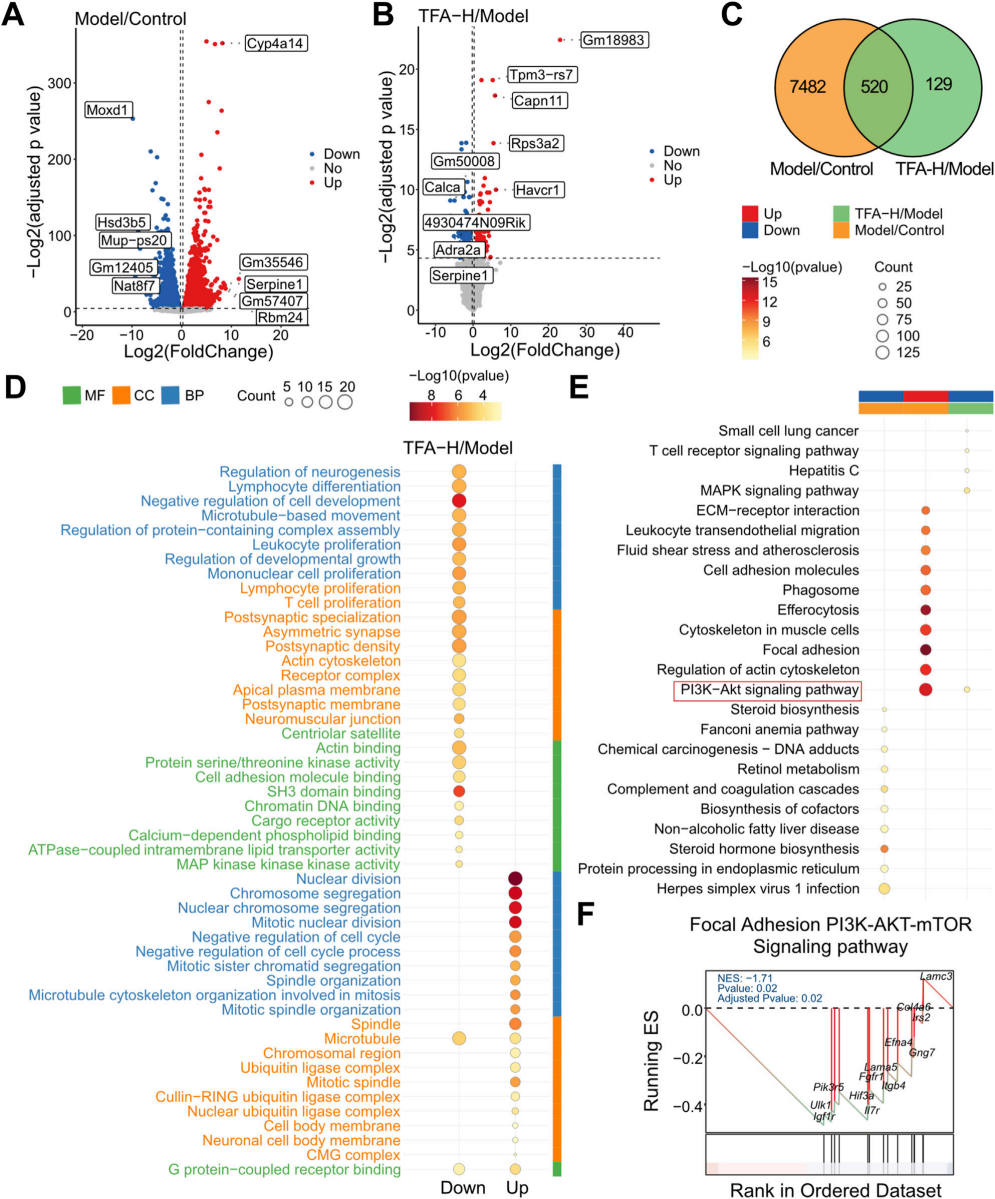
Transcriptomic analysis of TFA for MAFLD. **(A)** Differential genes in the normal and model groups. **(B)** Differential genes in the model and TFA-H groups. **(C)** Intersection analysis of differential genes by Wayne plot analysis. **(D)** GO analysis of differential genes in the model and TFA-H groups. **(E)** KEGG analysis of differentially expressed genes between the model and TFA-H groups. **(F)** GSEA analysis.

To further investigate the specific mechanisms by which TFA attenuates MAFLD, we performed transcriptome sequencing analyses to explore the molecular mechanisms. The differential genes among groups are presented in Figures 5A,B. When compared with the control group, 4,320 genes were upregulated and 3,682 genes were downregulated in the Model group. In contrast, relative to the Model group, 264 genes were over-expressed and 385 genes were under-expressed in the TFA-H group. Notably, 520 genes were identified as the common intersections among the three groups (Figure 5C). Subsequently, 649 differentially expressed genes between TFA-H and Model group, 8002 differentially expressed genes between Model and control group underwent Gene Ontology (GO), Kyoto Encyclopedia of Genes and Genomes (KEGG) pathway enrichment analyses. Additionally, Gene Set Enrichment Analysis (GSEA) was conducted on these genes using MsigDB canonical pathways. As depicted in Figure 5D, the GO analysis revealed significant enrichment in the following molecular functions: G protein-coupled receptor binding, protein serine/threonine kinase activity and cell adhesion molecule binding. Additionally, the KEGG pathway analysis demonstrated significant enrichment in the PI3K/AKT signalling pathway, MAPK signalling pathway and T cell receptor signalling pathway (Figure 5E). Meanwhile, GESA analysis showed significant enrichment in the PI3K/AKT/mTOR signalling pathway (Figure 5F).

### 3.6 TFA activates autophagy by modulating the PI3K/AKT/mTOR signalling pathway in liver tissue after treatment of MAFLD mice

**FIGURE 6:**
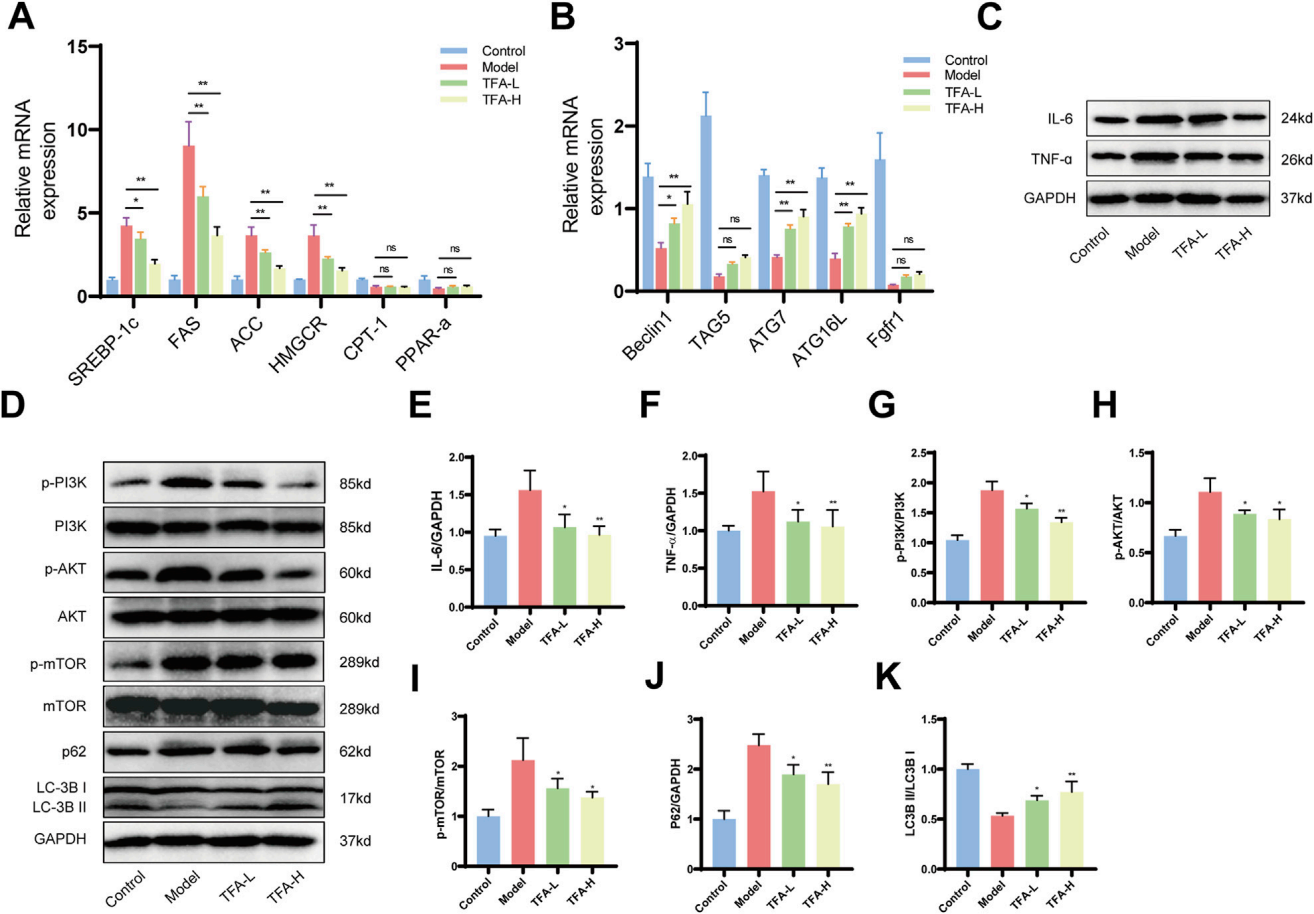
Effect of TFA on hepatic PI3K/AKT/mTOR signalling pathway and autophagy in MAFLD mice. **(A)** Effects of TFA on mRNA expression of genes related to lipid metabolism in mice liver. **(B)** Effects of TFA on mRNA expression of genes related to autophagy in mice liver. **(C)** Protein expression of IL-6 and TNF-α in mice liver. **(D)** Protein expression of p-PI3K, PI3K, p-AKT, AKT, p-mTOR, mTOR, LC-3B, and p62. **(E)** Relative protein quantification of IL-6. **(F)** Relative protein quantification of TNF-α. **(G)** Relative protein quantification of p-PI3K/PI3K. **(H)** Relative protein quantification of p-AKT/AKT. **(I)** Relative protein quantification of p-mTOR/mTOR. **(J)** Relative protein quantification of p62. **(K)** Relative protein quantification of LC3BII/I. ^*^ *P <* 0.05 vs. Model. ^**^ *P <* 0.01 vs. Model.

MAFLD is characterized by lipid metabolism disorders, inflammatory responses and progressive fibrosis. To elucidate the effects of TFA on these processes, we examined the expression of genes related to lipid metabolism. TFA-L and TFA-H primarily suppressed the expression of lipid synthesis genes, such as SREBP-1c, FAS, ACC and HMGCR, while genes involved in lipid catabolism, including CPT-1 and PPAR-α, remained unaffected (Figure 6A). To assess whether TFA promotes autophagy, we analyzed autophagy-related genes, including Beclin1, ATG7, and ATG16L, which were significantly upregulated in TFA-L and TFA-H group (Figure 6B).

In addition, TFA-L and TFA-H treatment downregulated hepatic inflammatory cytokine-related proteins, mitigating inflammation in MAFLD (Figures 6C,E,F). In lipid metabolism, The PI3K/AKT/mTOR signaling pathway, closely linked to autophagy, plays a pivotal role in regulating lipid metabolism and inflammation. Further investigation into the PI3K/AKT/mTOR pathway revealed elevated phosphorylation levels of PI3K, AKT, and mTOR in the model group. TFA-L and TFA-H intervention enhanced the LC3I-to-LC3II conversion rate and LC3II expression while reducing the autophagy substrate p62 protein levels (Figures 6D,G–K). These findings collectively demonstrate that TFA activates autophagy in the liver tissues of MAFLD mice, providing mechanistic insights into its therapeutic potential.

### 3.7 Effect of TFA on lipid deposition and inflammatory response in HepG2 cells

**FIGURE 7:**
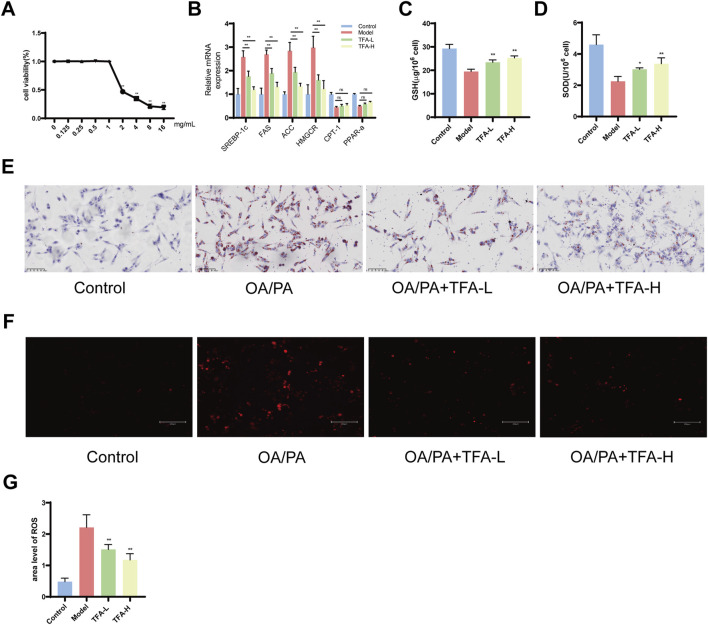
Effect of TFA on oxidative stress and inflammation levels in HepG2 cells. **(A)** Cell viability detected by CCK8. **(B)** The mRNA expression of genes related to lipid metabolism in HepG2 cells. **(C)** GSH content of HepG2 cells. **(D)** SOD content of HepG2 cells. **(E)** Oil red O staining. **(F)** ROS fluorescence staining. **(G)** ROS fluorescence area analysis. ^*^ *P <* 0.05 vs. Model. ^**^ *P <* 0.01 vs. Model.

Firstly, to ascertain the optimal concentration TFA on HepG2 cells, the CCK8 assay was employed to evaluate the effects of TFA at concentrations of 0, 0.125, 0.25, 0.5, 1, 2, 4, 8, and 16 mg/mL. Finally, a concentration of 0.5 mg/L (TFA-L group) and 1 mg/L (TFA-H group) was selected for the 24-h intervention as the subsequent treatment condition (Figure 7A). Subsequently, HepG2 cells were treated with a mixture of oleic acid (OA) and palmitic acid (PA) to establish the MAFLD cell model. To further examine the effects of TFA on lipid metabolism in HepG2 cells, we measured the expression of key lipid metabolism-related genes. The results showed that both TFA-L and TFA-H significantly downregulated key lipid synthesis-related genes, including SREBP-1c, FAS, ACC, and HMGCR. In contrast, TFA treatment had no significant effect on the expression of fatty acid oxidation-related genes (CPT-1 and PPAR-α) (Figure 7B). Meanwhile, TFA-L and TFA-H increased the levels of GSH and SOD in the cells (Figures 7C,D).

The Oil Red O staining demonstrated that after 24 h of treatment, TFA-L and TFA-H significantly mitigated lipid deposition (Figure 7E). Moreover, the ROS staining of HepG2 cells revealed that TFA-L and TFA-H remarkably decreased the levels of reactive oxygen species (Figures 7F,G). Collectively, the above-mentioned results indicated that TFA-L and TFA-H could reduce lipid deposition and oxidative stress damage in HepG2 cells.

### 3.8 TFA regulates the PI3K/AKT/mTOR signalling pathway to enhance autophagy in HepG2 cells

**FIGURE 8:**
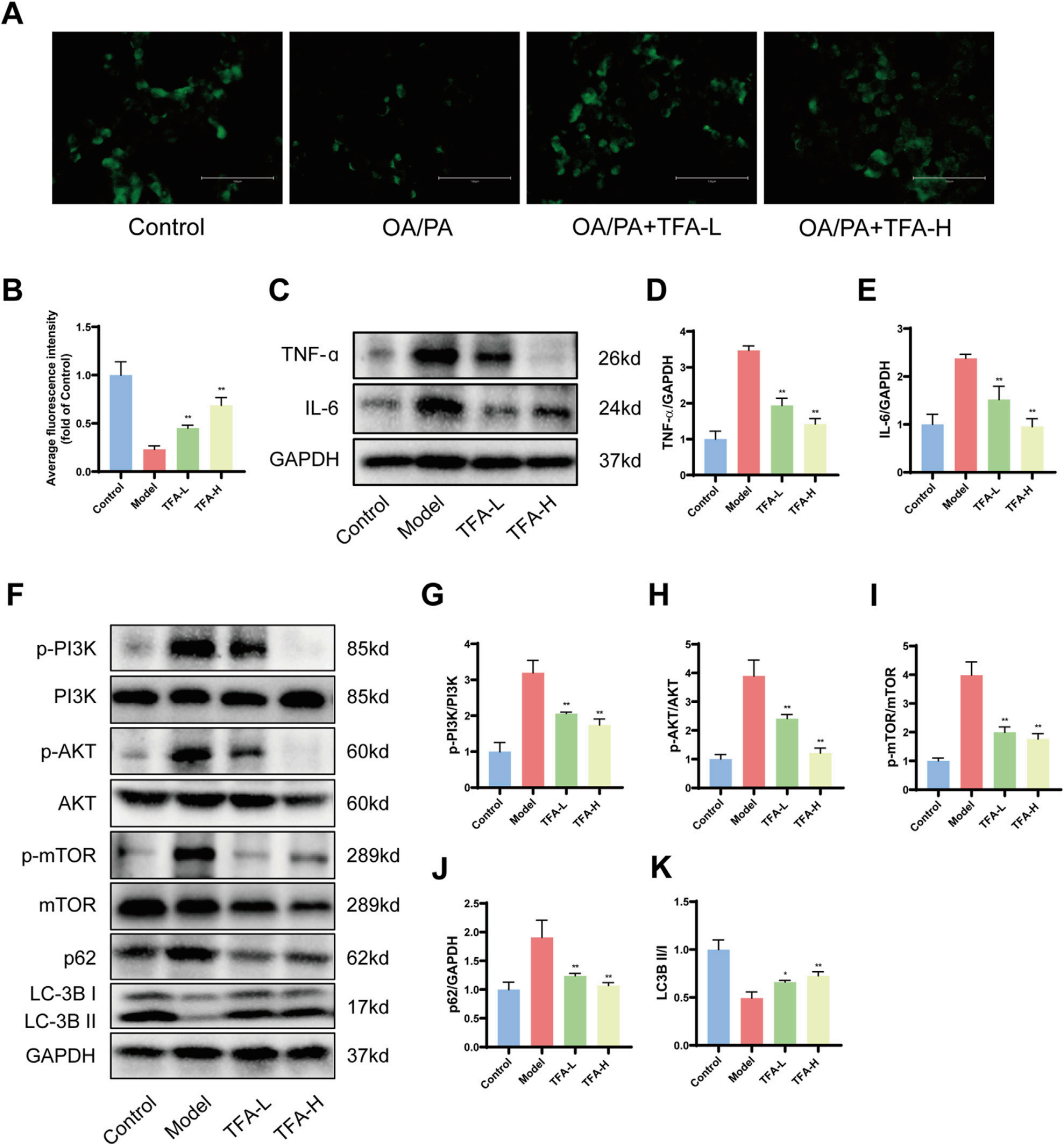
Effect of TFA on PI3K/AKT/mTOR signalling pathway and autophagy in HepG2 cells. **(A)** MDC fluorescence staining. **(B)** Average fluorescence intensity of MDC. **(C)** Protein expression of IL-6 and TNF-α in HepG2 cells. **(D)** Relative quantification of TNF-α. **(E)** Relative quantification of IL-6. **(F)** Protein expression of p-PI3K, PI3K, p-AKT, AKT, p-mTOR, mTOR, LC-3B, and p62 protein expression. **(G)** Relative quantification of p-PI3K/PI3K. **(H)** Relative quantification of p-AKT/AKT. **(I)** Relative protein quantification of p-mTOR/mTOR. **(J)** Relative protein quantification of p62. **(K)** Relative protein quantification of LC3BII/I. ^*^ *P <* 0.05 vs. Model. ^**^ *P <* 0.01 vs. Model.

We investigated the effects of TFA-L and TFA-H on autophagy in HepG2 cells using MDC as a fluorescent probe. With increasing drug concentrations, we observed a dose-dependent increase in autophagic vesicles compared to the model group (Figure 8A).

Following a 24 h intervention with OA and PA in HepG2 cells, the expression of IL-6, TNF-α, p-PI3K, p-AKT, and p-mTOR proteins was significantly upregulated in the model group. However, after 24 h of TFA treatment, the levels of IL-6, TNF-α, p-PI3K, p-AKT, and p-mTOR proteins were notably decreased. Upon TFA intervention, the expression of LC3II protein and the LC3II/I ratio were enhanced, while the expression of p62 protein was significantly reduced (Figures 8B–K). These findings indicated that the PI3K/AKT/mTOR signaling pathway was implicated in the treatment of MAFLD by TFA.

### 3.9 Inhibition of autophagy eliminates the protective effect of TFA on HepG2 cells

**FIGURE 9:**
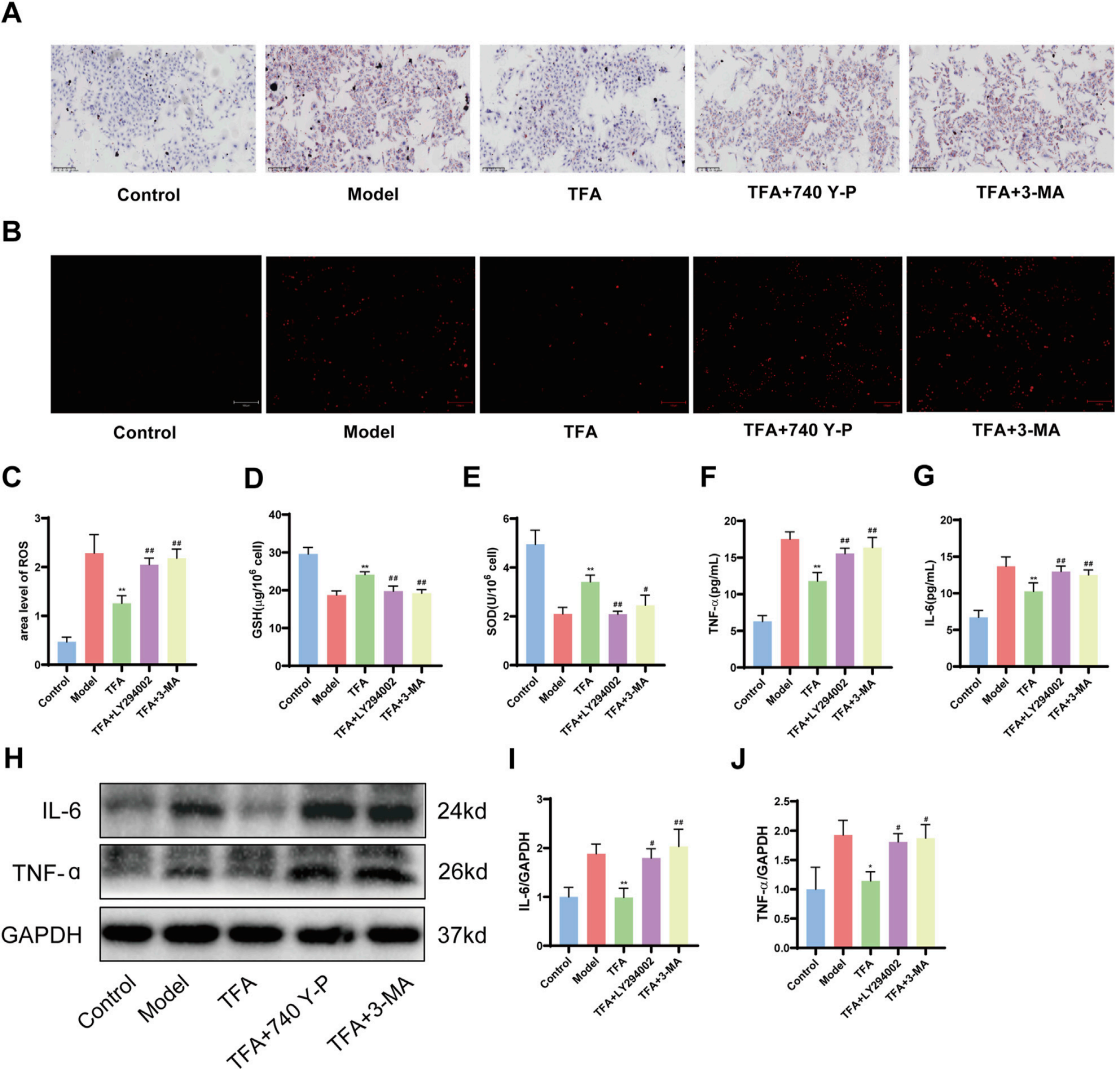
Effect of 740 Y-P (PI3K agonist) and 3-MA (autophagy inhibitor) on the protective effect of TFA. **(A)** Oil red O staining. **(B)** ROS fluorescence staining. **(C)** ROS fluorescence area analysis. **(D)** GSH content of HepG2 cells. **(E)** SOD content of HepG2 cells. **(F)** IL-6 level of HepG2 cell supernatants. **(G)** TNF-α level of HepG2 cell supernatants. **(H)** Protein expression of IL-6 and TNF-α in HepG2 cells. **(I)** Relative quantification of IL-6. **(J)** Relative quantification of TNF-α. ^*^ *P <* 0.05 vs. Model. ^**^ *P <* 0.01 vs. Model.

In order to explore the potential pivotal roles of autophagy and the PI3K/AKT/mTOR signaling pathway in the protective mechanism of TFA against MAFLD, a series of experimental interventions were carried out. OA/PA treated HepG2 cells were further exposed to the autophagy inhibitor 3-MA (5 mM) and the PI3K agonist 740 Y-P (20 μM) either in the presence or absence of TFA (Wang et al., 2024; Yan et al., 2022). As hypothesized, the co-addition of 3-MA and 740 Y-P exerted a significant inhibitory effect on the beneficial actions of TFA. Specifically, this led to a notable elevation in lipid accumulation within HepG2 cells (Figure 9A). Through the systematic assessment of ROS staining and the quantification of GSH and SOD levels, it was clearly demonstrated that the combined treatment with 3-MA and 740 Y-P markedly suppressed the antioxidant effects conferred by TFA (Figures 9B–E). To further analyze changes in inflammatory levels, we measured IL-6 and TNF-α levels in the cell supernatant using ELISA and assessed their protein expression in cells via western blot. The results revealed that the addition of 740 Y-P and 3-MA significantly attenuated the protective effects of TFA (Figures 9F–J).

## 4 Discussion

The global prevalence of MAFLD continues to rise, yet the development of clinically validated therapeutic interventions remains an unmet medical need. MAFLD pathogenesis encompasses a multifactorial interplay of molecular mechanisms, including hepatic insulin resistance, impaired lipid homeostasis, mitochondrial oxidative stress, chronic inflammation, and gut-liver axis dysregulation. (Friedman et al., 2018; Xu et al., 2022). The multifactorial nature of MAFLD pathophysiology limits the therapeutic efficacy of single-target pharmacological approaches, necessitating the development of multi-modal intervention strategies. Consequently, there is growing emphasis on the development of pleiotropic therapeutic agents capable of simultaneously modulating multiple pathological pathways in MAFLD management. Phytochemicals have emerged as promising therapeutic candidates for MAFLD, owing to their inherent ability to modulate multiple molecular targets through synergistic mechanisms of action. Preclinical studies have demonstrated that bioactive metabolites, particularly flavonoids (e.g., quercetin), polyphenols (e.g., epigallocatechin gallate), and terpenoids (e.g., ursolic acid), exert hepatoprotective effects through coordinated regulation of lipid homeostasis, antioxidant defense systems, and inflammatory signaling pathways (He et al., 2023; Yu et al., 2024). Specifically, resveratrol has been shown to enhance insulin receptor substrate-1 phosphorylation, while curcumin modulates AMP-activated protein kinase signaling, both demonstrating significant attenuation of hepatic lipid accumulation in preclinical models (Ding et al., 2018; Yan et al., 2018). Elucidating the molecular mechanisms underlying the therapeutic effects of these phytochemicals not only advances our understanding of MAFLD pathophysiology but also informs the rational design of next-generation multi-target therapeutic agents. Future research directions should prioritize the systematic characterization of molecular signaling networks modulated by these metabolites, coupled with rigorous evaluation of their clinical efficacy through well-designed randomized controlled trials, to facilitate the translation of these findings into evidence-based MAFLD therapies.

The advent of ultra-high-performance liquid chromatography coupled with UHPLC-Q-Orbitrap HRMS has transformed the analysis of TCM matrices, achieving sub-ppm mass accuracy and enabling comprehensive metabolite profiling (Liu R. et al., 2020; Liu Z. et al., 2024; Bi et al., 2021). Employing UHPLC-Q-Orbitrap HRMS, we conducted a systematic chemical profiling of TFA, acquiring high-resolution MS/MS spectra in both positive and negative ionization modes to establish its comprehensive phytochemical inventory. A total of 56 metabolites were identified in TFA, primarily comprising flavonoids, coumarins and their derivatives, organic oxygen compounds, isoprenoid lipids, cinnamic acids and their derivatives, as well as benzene and its substituted derivatives. Notably, several metabolites with established efficacy in treating MAFLD were identified, including hyperoside, rutin, isoquercitrin, quercetin-3-O-β-D-glucopyranoside, and myricetin 3-O-glucoside (Wang, Sheng, 2021; Liu Y. et al., 2024; Yi et al., 2023; Jin et al., 2024). These flavonoids were identified as bioactive substances with potent anti-inflammatory and antioxidant properties. Specifically, hyperoside has been shown to ameliorate MAFLD by regulating cholesterol metabolism and bile acid metabolism and excretion (Wang, Sheng, 2021; Jang, 2022). Additionally, rutin can impede the progression of MAFLD by alleviating inflammation and upregulating the expression of genes related to fatty acid oxidation (Liu Y. et al., 2024; Tung et al., 2021). These findings not only elucidate the pharmacological activities of the major metabolites in TFA but also provide a robust theoretical basis for its potential application in the treatment of MAFLD.

The pathological progression of MAFLD is intricately linked to chronic inflammation and progressive fibrosis, driven by the activation of pro-inflammatory cytokines and extracellular matrix deposition (Chen and Zhao, 2024; Dong et al., 2023). Excessive inflammation, characterized by elevated levels of cytokines such as TNF-α and IL-6, and oxidative stress, marked by increased ROS and MDA, significantly contribute to hepatocyte damage and the progression to more severe liver conditions, including non-alcoholic steatohepatitis and cirrhosis (Clare et al., 2022; Qu et al., 2021). Our data demonstrate that TFA treatment significantly reduced hepatic inflammation and fibrosis, while enhancing antioxidant capacity in MAFLD mice. These results underscore the potential of TFA as a multi-target therapeutic agent for MAFLD, effectively addressing key pathological mechanisms, including lipid accumulation, inflammation, and oxidative stress. The multifaceted actions of TFA, targeting multiple pathways, make it a promising candidate for the development of novel, integrative therapies for MAFLD. Excessive lipid accumulation activates pro-inflammatory signaling in hepatocytes and recruits immune cells, thereby amplifying the inflammatory cascade (Wei et al., 2025; Zhao et al., 2024). The anti-inflammatory properties of TFA in hepatocytes may break this vicious cycle, potentially offering therapeutic benefits for metabolic dysfunction-associated steatotic liver disease.

Untargeted metabolomics analysis identified significant perturbations in hepatic metabolic pathways, particularly nucleotide metabolism, pyrimidine metabolism, central carbon metabolism, and D-amino acid metabolism in MAFLD progression. Dysregulation of nucleotide metabolism can lead to an energy imbalance in hepatocytes, impairing liver repair and regenerative capacity, and exacerbating hepatic injury and inflammatory responses (Caddeo and Romeo, 2024). The AKT/mTORC1 signaling pathway promotes pyrimidine and purine synthesis by activating ribonucleotide reductase M2. Furthermore, this pathway modulates one-carbon metabolism (folate cycle) to provide methyl donors required for nucleotide biosynthesis. Disruption of pyrimidine metabolism, further promotes hepatocyte damage and fibrotic progression (Petta et al., 2024). Aberrations in central carbon metabolism, such as increased lactate production and decreased NAD^+^/NADH ratios, result in metabolic energy dysfunction, promoting lipid accumulation and oxidative stress, thereby aggravating hepatic inflammation and fibrosis (Patterson et al., 2016). Dysregulation of D-amino acid metabolism, disrupts hepatic metabolic homeostasis, contributing to lipid metabolic disorders and hepatocyte injury (Demirel et al., 2023). Following TFA treatment, the number of differential metabolites was significantly reduced, indicating that TFA partially restored metabolic balance in the livers of MAFLD mice. Improvements in lipid and lipid-like molecule metabolism, including reduced triglyceride content and increased phospholipid levels, suggest that TFA alleviates hepatic steatosis by modulating lipid metabolic pathways. Enrichment analysis of nucleotide and pyrimidine metabolism pathways demonstrated that TFA mitigates MAFLD-associated hepatocyte injury by restoring pyrimidine metabolism and nucleotide metabolism.

The PI3K/AKT/mTOR axis serves as a central regulator of hepatic autophagy flux, with its dysregulation significantly contributing to MAFLD progression (Fan X. et al., 2023; Huang et al., 2021; Alshehade et al., 2022). Mechanistically, mTORC1 suppressing autophagosome initiation complex assembly (Kim and Guan, 2015). In MAFLD, hyperactivated PI3K/AKT signaling sustains mTORC1 activity, resulting in autophagic flux impairment and hepatic lipotoxicity (Lin et al., 2025; Chen and Lin, 2022; Wang et al., 2022). Activation of the PI3K/AKT signaling pathway enhances mTOR activity, which in turn suppresses autophagy, leading to increased hepatic lipid accumulation, insulin resistance, and inflammation. Conversely, inhibition of this pathway promotes autophagy, facilitating the degradation of lipid droplets and alleviating hepatic steatosis (Zhao et al., 2021). Recent studies have demonstrated that modulating the PI3K/AKT/mTOR axis can restore autophagic flux, improve lipid metabolism, and mitigate liver injury in MAFLD models (Tsuji et al., 2023). Transcriptomic profiling in this study suggests that TFA’s therapeutic efficacy in MAFLD may be mediated through modulation of the PI3K/AKT signaling pathway. This was corroborated by *in vivo* and *in vitro* experiments showing that TFA significantly affects critical metabolites of the PI3K/AKT/mTOR pathway, thereby reestablishing autophagic function and alleviating MAFLD-associated pathologies.

Specifically, TFA treatment was shown to enhance the expression of autophagy-related genes, such as Beclin1, ATG7, and ATG16L, while reducing the phosphorylation levels of PI3K, AKT, and mTOR (Sundarraj et al., 2021). These molecular changes were accompanied by a significant increase in the LC3I-to-LC3II conversion ratio and a marked decrease in the levels of the autophagy substrate p62, indicative of enhanced autophagic flux (Kaizuka et al., 2016). By restoring autophagy, TFA effectively reduced lipid accumulation, suppressed inflammatory responses and attenuated fibrosis in MAFLD models. These findings provide robust experimental evidence and detailed molecular insights into the therapeutic potential of TFA for MAFLD, highlighting its multifaceted effects on lipid metabolism, inflammation, and fibrosis. Targeting the PI3K/AKT/mTOR pathway and its associated autophagic processes represents a promising strategy for developing multi-target therapies for MAFLD, addressing the complex and multifactorial nature of the disease. Further preclinical and clinical studies are warranted to explore the long-term efficacy, safety, and clinical applicability of TFA in the treatment of MAFLD.

## 5 Conclusion

**FIGURE 10:**
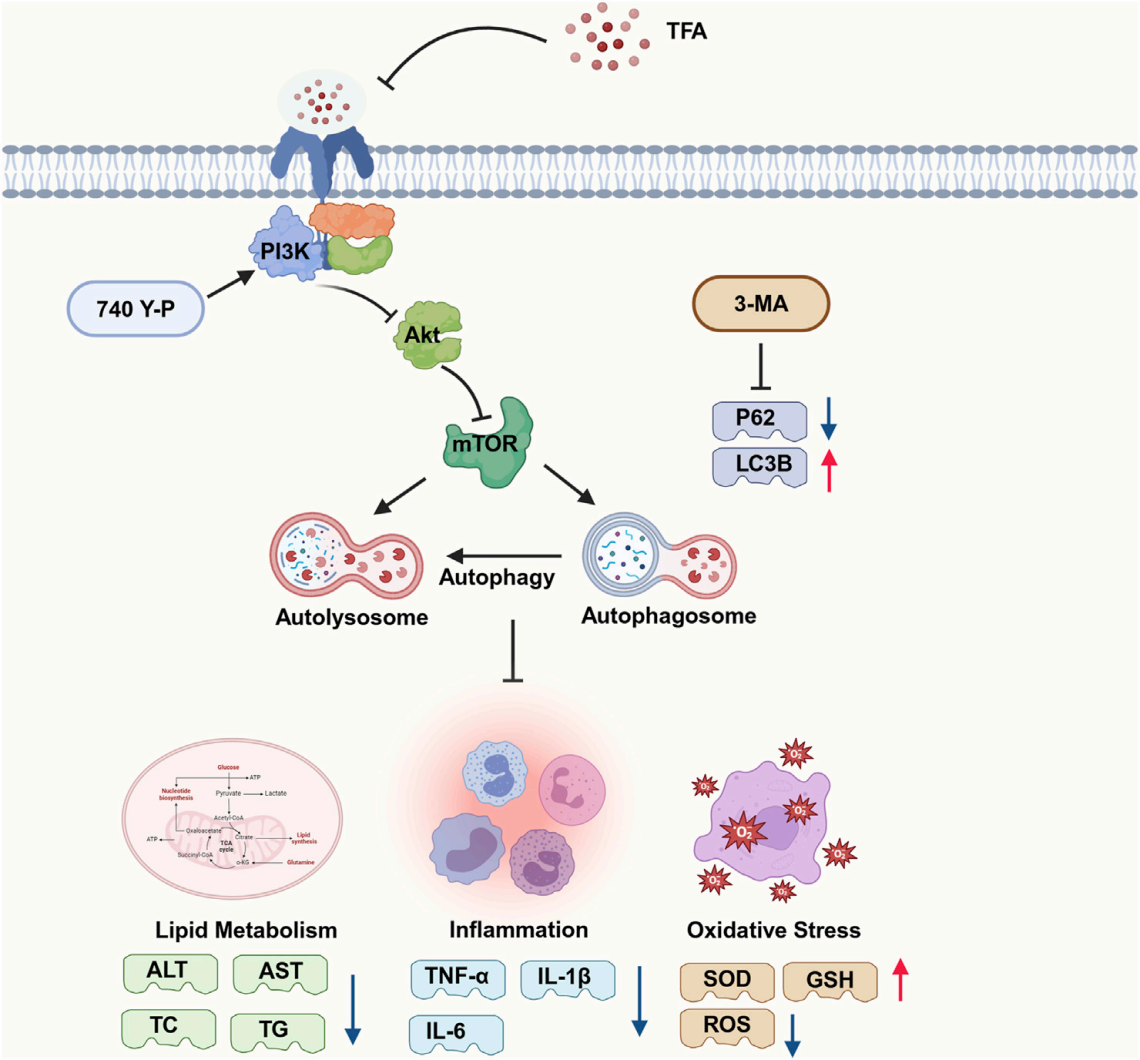
Mechanism of action of TFA in the treatment of MAFLD. Part of the picture provided by Biorender. TFA may activate autophagy by inhibiting PI3K/AKT/mTOR signaling pathway, thereby reducing lipid deposition and the release of inflammatory factors and achieving the goal of treating MAFLD. 3-MA inhibition of autophagy can downregulated the levels of SOD and GSH and upregulated the expression levels of IL-1β and IL-6. These results prove that the activation of autophagy is inversely related to the regulation of lipid deposition and inflammatory response.

Our study elucidates that TFA exerts its therapeutic effects on MAFLD by targeting the PI3K/AKT/mTOR signaling cascade, which leads to the induction of autophagy, ultimately enhancing lipid homeostasis, mitigating inflammatory responses, and alleviating oxidative damage (Figure 10). The results underscore the potential of TFA as a multi-target therapeutic strategy for MAFLD, offering a rationale for further clinical investigations and drug development efforts.

## Data Availability

The original contributions presented in the study are publicly available. This data can be found here: https://doi.org/10.6084/m9.figshare.29436344.v3.

## Abbreviations

ALT, Alanine aminotransferase; AST, Aspartate aminotransferase; α-SMA, α-smooth muscle actin; Col-1, Type I collagen; ELISA, Enzyme Linked Immunosorbent Assay; FBS, Fetal bovine serum; GO, Gene Ontology; HDL-C, High-density lipoprotein cholesterol; HE, Hematoxylin and eosin; HFD, High-fat diet; KEGG, Kyoto Encyclopedia of Genes and Genomes; LC-MS, Liquid chromatography-mass spectrometry; LDL-C, Low-density lipoprotein cholesterol; MAFLD, Metabolic Associated Fatty Liver Disease; NCD, Normal chow diet; OA, leic acid; ORO, Oil Red O; PA, Palmitic acid; PBS, Phosphate-buffered saline; RT-qPCR, Real-Time Quantitative Reverse Transcription Polymerase Chain Reaction; TC, Total cholesterol; TFA-H, Total flavonoids from Abelmoschus manihot high-dose; TFA-L, Total flavonoids from Abelmoschus manihot low-dose; TG, Triglycerides; UHPLC-Q-Orbitrap HRMS, Ultra-high-performance liquid chromatography-quadrupole orbitrap high-resolution mass spectrometry; WB, Western blot.
