# Supplementary material for: Multi-omics reveals total flavones from Abelmoschus manihot (L.) Medik. [Malvaceae] ameliorate MAFLD via PI3K/AKT/mTOR-mediated autophagy
Source: Front Pharmacol. 2025 Jul 11;16:1601707. doi: 10.3389/fphar.2025.1601707 (PMC12289637; doi:10.3389/fphar.2025.1601707)
Supplement: Supplementary file 1 [file Table1.docx]

**Supplementary Table 1 The primers of relative gene**

| **Gene** | **Primer** |
| --- | --- |
| Beclin1 | FP:ACCTCAGCCGAAGACTGAAG |
|  | RP:AACAGCGTTTGTAGTTCTGACA |
| ATG7 | FP:ATGATCCCTGTAACTTAGCCCA |
|  | RP:CACGGAAGCAAACAACTTCAAC |
| ATG16L | FP:TCTGGGACATTCGATCAGAGAG |
|  | RP:CCTTTCTGGGTTTAAGTCCAGG |
| TAG5 | FP:CTTGCATCAAGTTCAGCTCTTCC |
|  | RP:AAGTGAGCCTCAACCGCATCCT |
| FGFR1 | FP:CCCGTAGCTCCATATTGGACA |
|  | RP:TTTGCCATTTTTCAACCAGCG |
| SREBP-1c | FP:ACAGTGACTTCCCTGGCCTAT |
|  | RP:GCATGGACGGGTACATCTTCAA |
| FAS | FP:TCTGGTTCTTACGTCTGTTGC |
|  | RP:CTGTGCAGTCCCTAGCTTTCC |
| ACC | FP:ATGTCTGGCTTGCACCTAGTA |
|  | RP:CCCCAAAGCGAGTAACAAATTCT |
| HMGCR | FP:TGATTGACCTTTCCAGAGCAAG |
|  | RP:CTAAAATTGCCATTCCACGAGC |
| CPT-1 | FP:ATCAATCGGACTCTGGAAACGG |
|  | RP:TCAGGGAGTAGCGCATGGT |
| PPAR-a | FP:ATGGTGGACACGGAAAGCC |
|  | RP:CGATGGATTGCGAAATCTCTTGG |
| GAPDH | FP:GGAGCGAGATCCCTCCAAAAT |
|  | RP:GGCTGTTGTCATACTTCTCATGG |
