# Supplementary material for: Multi-omics reveals total flavones from Abelmoschus manihot (L.) Medik. [Malvaceae] ameliorate MAFLD via PI3K/AKT/mTOR-mediated autophagy
Source: Front Pharmacol. 2025 Jul 11;16:1601707. doi: 10.3389/fphar.2025.1601707 (PMC12289637; doi:10.3389/fphar.2025.1601707)
Supplement: Supplementary file 2 [file Table2.docx]

**Supplementary Table 2 The 56 chemical components of TFA based on UHPLC-Q-Orbitrap HRMS**

| **No.** | **Ion Mod** | **Retention time (min)** | **English Name** | **Formula** | **Experimental m/z** | **Theoretical m/z** | **Mass Error (ppm)** | **Fragment Score** | **class** | **subclass** |
| --- | --- | --- | --- | --- | --- | --- | --- | --- | --- | --- |
| **1** | POS-NEG | 11.07 | Hyperoside | C21H20O12 | 465.1033 | 465.1028 | 1.03 | 96.70 | Flavonoids | Flavonoid glycosides |
| **2** | POS-NEG | 10.84 | Rutin | C27H30O16 | 611.1612 | 611.1607 | 0.81 | 71.60 | Flavonoids | Flavonoid glycosides |
| **3** | POS | 11.31 | Isoquercitrin | C21H20O12 | 465.1035 | 465.1028 | 1.53 | 96.30 | Flavonoids | Flavonoid glycosides |
| **4** | POS-NEG | 13.40 | Quercetin -3-o-β-d-glucopyranoside | C21H20O12 | 465.1035 | 465.1028 | 1.45 | 73.50 | Flavonoids | Flavonoid glycosides |
| **5** | POS-NEG | 10.09 | Myricetin 3 -o-glucoside | C21H20O13 | 481.0983 | 481.0977 | 1.21 | 70.60 | Flavonoids | Flavonoid glycosides |
| **6** | NEG | 11.49 | Cannabiscitrin | C21H20O13 | 479.0832 | 479.0831 | 0.29 | 63.90 | Flavonoids | Flavonoid glycosides |
| **7** | POS-NEG | 13.06 | Myricetin | C15H10O8 | 317.0302 | 317.0303 | -0.26 | 55.90 | Flavonoids | Flavones |
| **8** | POS-NEG | 15.74 | Tiliroside | C30H26O13 | 593.1306 | 593.1300 | 0.89 | 90.60 | Flavonoids | Flavonoid glycosides |
| **9** | POS-NEG | 9.83 | Hibiscetin -3-o-glucoside | C21H20O14 | 495.0783 | 495.0780 | 0.59 | 59.20 | - | - |
| **10** | POS-NEG | 11.67 | Nicotiflorin | C27H30O15 | 593.1519 | 593.1512 | 1.16 | 69.20 | Flavonoids | Flavonoid glycosides |
| **No.** | **Ion Mod** | **Retention time (min)** | **English Name** | **Formula** | **Experimental m/z** | **Theoretical m/z** | **Mass Error (ppm)** | **Fragment Score** | **class** | **subclass** |
| **11** | POS-NEG | 12.02 | Astragalin | C21H20O11 | 447.0938 | 447.0933 | 1.21 | 91.30 | Flavonoids | Flavonoid glycosides |
| **12** | NEG | 10.91 | Ellagic acid | C14H6O8 | 300.9989 | 300.9990 | -0.24 | 26.70 | Tannins | Hydrolyzable tannins |
| **13** | POS | 11.66 | Cynaroside | C21H20O11 | 449.1089 | 449.1079 | 2.21 | 88.90 | Flavonoids | Flavonoid glycosides |
| **14** | POS | 14.74 | Hyperin | C21H19O12- | 927.1838 | 464.0950 | 997832.02 | 52.90 | Flavonoids | Flavonoid glycosides |
| **15** | POS | 9.34 | Fraxetin | C10H8O5 | 209.0447 | 209.0445 | 1.19 | 86.90 | Coumarins and derivatives | Hydroxycoumarins |
| **16** | NEG | 7.44 | Chlorogenic acid | C16H18O9 | 353.0881 | 353.0878 | 0.96 | 79.50 | Organooxygen compounds | Alcohols and polyols |
| **17** | POS-NEG | 17.17 | Kaempferol | C15H10O6 | 287.0552 | 287.0550 | 0.52 | 76.70 | Flavonoids | Flavones |
| **18** | NEG | 8.36 | Fraxin | C16H18O10 | 369.0828 | 369.0827 | 0.35 | 65.10 | Coumarins and derivatives | Coumarin glycosides |
| **19** | POS | 6.31 | 3,4-dihydroxycinnamic acid | C9H8O4 | 163.0393 | 163.0390 | 2.07 | 32.60 | Cinnamic acids and derivatives | Hydroxycinnamic acids and derivatives |
| **20** | POS-NEG | 6.53 | Esculin | C15H16O9 | 339.0724 | 339.0721 | 0.69 | 85.00 | Coumarins and derivatives | Coumarin glycosides |
| **No.** | **Ion Mod** | **Retention time (min)** | **English Name** | **Formula** | **Experimental m/z** | **Theoretical m/z** | **Mass Error (ppm)** | **Fragment Score** | **class** | **subclass** |
| **21** | POS-NEG | 8.75 | L-epicatechin | C15H14O6 | 291.0864 | 291.0863 | 0.26 | 82.60 | Flavonoids | Flavans |
| **22** | POS | 10.86 | Scopoletin | C10H8O4 | 193.0499 | 193.0496 | 1.53 | 80.70 | Coumarins and derivatives | Hydroxycoumarins |
| **23** | POS-NEG | 6.79 | (-)-epigallocatechin | C15H14O7 | 305.0670 | 305.0667 | 1.06 | 86.50 | Flavonoids | Flavans |
| **24** | POS-NEG | 8.38 | Quercetin -3-o-robinobioside | C27H30O16 | 609.1473 | 609.1461 | 2.07 | 74.20 | Flavonoids | Flavonoid glycosides |
| **25** | POS | 14.92 | Luteolin | C15H10O6 | 287.0553 | 287.0550 | 0.77 | 40.20 | Flavonoids | Flavones |
| **26** | POS | 7.80 | 6-methoxyl -7-hydroxycoumarin | C10H8O4 | 193.0500 | 193.0496 | 2.37 | 31.00 | Coumarins and derivatives | Hydroxycoumarins |
| **27** | POS-NEG | 16.80 | Naringenin | C15H12O5 | 271.0614 | 271.0612 | 0.72 | 82.70 | Flavonoids | Flavans |
| **28** | POS-NEG | 10.60 | Typhaneoside | C34H42O20 | 769.2207 | 769.2197 | 1.33 | 79.60 | Flavonoids | Flavonoid glycosides |
| **29** | POS | 9.78 | P-coumaric acid | C9H8O3 | 147.0443 | 147.0440 | 1.61 | 71.90 | Cinnamic acids and derivatives | Hydroxycinnamic acids and derivatives |
| **30** | NEG | 17.75 | Baicalein | C15H10O5 | 269.0456 | 269.0455 | 0.42 | 87.90 | Flavonoids | Flavones |
| **No.** | **Ion Mod** | **Retention time (min)** | **English Name** | **Formula** | **Experimental m/z** | **Theoretical m/z** | **Mass Error (ppm)** | **Fragment Score** | **class** | **subclass** |
| **31** | POS | 10.88 | Ferulic acid | C10H10O4 | 177.0549 | 177.0546 | 1.40 | 87.30 | Cinnamic acids and derivatives | Hydroxycinnamic acids and derivatives |
| **32** | POS | 11.44 | Isofraxidin | C11H10O5 | 223.0604 | 223.0601 | 1.02 | 73.60 | Coumarins and derivatives | Hydroxycoumarins |
| **33** | POS | 4.66 | Protocatechuic acid | C7H6O4 | 155.0343 | 155.0339 | 2.66 | 77.80 | Benzene and substituted derivatives | Benzoic acids and derivatives |
| **34** | POS-NEG | 7.24 | Catechin | C15H14O6 | 289.0721 | 289.0717 | 1.09 | 71.90 | Flavonoids | Flavans |
| **35** | NEG | 6.02 | Salicin | C13H18O7 | 331.1038 | 331.1035 | 0.99 | 21.90 | Organooxygen compounds | Carbohydrates and carbohydrate conjugates |
| **36** | POS | 11.14 | Sinapic acid | C11H12O5 | 207.0654 | 207.0652 | 1.14 | 76.60 | Cinnamic acids and derivatives | Hydroxycinnamic acids and derivatives |
| **37** | POS | 8.77 | Puerarin | C21H20O9 | 417.1184 | 417.1180 | 0.76 | 89.30 | Isoflavonoids | Isoflavonoid C-glycosides |
| **38** | NEG | 8.38 | Geniposide | C17H24O10 | 387.1301 | 387.1297 | 1.22 | 56.80 | Prenol lipids | Terpene glycosides |
| **No.** | **Ion Mod** | **Retention time (min)** | **English Name** | **Formula** | **Experimental m/z** | **Theoretical m/z** | **Mass Error (ppm)** | **Fragment Score** | **class** | **subclass** |
| **39** | NEG | 5.03 | Helicid | C13H16O7 | 329.0880 | 329.0878 | 0.69 | 24.50 | Organooxygen compounds | Carbohydrates and carbohydrate conjugates |
| **40** | POS | 7.80 | Syringin | C17H24O9 | 390.1766 | 390.1758 | 1.93 | 40.10 | Organooxygen compounds | Carbohydrates and carbohydrate conjugates |
| **41** | POS | 16.40 | Pinoresinol | C20H22O6 | 341.1389 | 341.1383 | 1.74 | 75.80 | Furanoid lignans | - |
| **42** | POS-NEG | 8.83 | Quercetin-3-robinobioside | C27H30O16 | 609.1472 | 609.1461 | 1.80 | 59.30 | Flavonoids | Flavonoid glycosides |
| **43** | NEG | 5.56 | 6-alpha-hydroxygeniposide | C17H24O11 | 449.1305 | 449.1301 | 1.00 | 61.20 | Prenol lipids | Terpene glycosides |
| **44** | POS | 9.65 | Isovanilline | C8H8O3 | 153.0549 | 153.0546 | 1.99 | 72.90 | Phenols | Methoxyphenols |
| **45** | POS | 8.62 | Syringic acid | C9H10O5 | 199.0604 | 199.0601 | 1.15 | 26.50 | Benzene and substituted derivatives | Benzoic acids and derivatives |
| **46** | POS | 7.80 | Esculetin | C9H6O4 | 179.0342 | 179.0339 | 1.41 | 62.40 | Coumarins and derivatives | Hydroxycoumarins |
| **47** | POS | 9.84 | 7-hydroxycoumarin | C9H6O3 | 163.0392 | 163.0390 | 1.09 | 33.00 | Coumarins and derivatives | Hydroxycoumarins |
| **No.** | **Ion Mod** | **Retention time (min)** | **English Name** | **Formula** | **Experimental m/z** | **Theoretical m/z** | **Mass Error (ppm)** | **Fragment Score** | **class** | **subclass** |
| **48** | NEG | 10.82 | Piceid | C20H22O8 | 435.1296 | 435.1297 | -0.13 | 80.40 | Stilbenes | Stilbene glycosides |
| **49** | POS | 6.15 | 3,4-dihydroxybenzaldehyde | C7H6O3 | 139.0394 | 139.0390 | 2.66 | 68.20 | Organooxygen compounds | Carbonyl compounds |
| **50** | POS | 8.04 | Isovanillic acid | C8H8O4 | 169.0498 | 169.0496 | 1.63 | 52.50 | Benzene and substituted derivatives | Benzoic acids and derivatives |
| **51** | POS | 9.45 | 4-hydroxyacetophenone | C8H8O2 | 137.0600 | 137.0597 | 2.09 | 45.80 | Organooxygen compounds | Carbonyl compounds |
| **52** | NEG | 17.77 | Dihydroartemisinin | C15H24O5 | 283.1552 | 283.1551 | 0.37 | 59.70 | Prenol lipids | Sesquiterpenoids |
| **53** | NEG | 15.86 | Andrographolide | C20H30O5 | 349.2023 | 349.2020 | 0.87 | 21.30 | Lactones | Gamma butyrolactones |
| **54** | NEG | 22.95 | Β-eudesmol | C15H28O | 269.2123 | 269.2122 | 0.31 | 46.80 | Prenol lipids | Sesquiterpenoids |
| **55** | NEG | 21.83 | Isoalantolactone | C15H20O2 | 277.1445 | 277.1445 | -0.08 | 50.30 | Prenol lipids | Terpene lactones |
| **56** | POS | 8.12 | Daphnetin | C9H6O4 | 179.0342 | 179.0339 | 1.57 | 28.90 | Coumarins and derivatives | Hydroxycoumarins |
